# *MyD88* Polymorphisms and Association with Susceptibility to Salmonella Pullorum

**DOI:** 10.1155/2015/692973

**Published:** 2015-12-31

**Authors:** Xian-Qing Liu, Fei Wang, Jie Jin, Yu-Guang Zhou, Jin-Shan Ran, Ze-Qing Feng, Yan Wang, Yi-Ping Liu

**Affiliations:** Farm Animal Genetic Resources Exploration and Innovation Key Laboratory of Sichuan Province, Sichuan Agricultural University, Chengdu Campus, Chengdu 611130, China

## Abstract

Myeloid differentiation primary response gene 88 (MYD88), a universal adapter protein, plays an important role in activating the nuclear factor-*κ*B (NF-*κ*B) and regulating the expression of proinflammatory genes like tumor necrosis factor (TNF) and interleukin-1 (IL-1), which were highly involved in Salmonella Pullorum infection. To detect the relationship between polymorphisms of the* MyD88* gene and Salmonella Pullorum disease, we screened the coding region (CDS) of the* MYD88* gene by DNA pool construction and sequencing based on case-control study. Eight single nucleotide polymorphisms (SNPs) in the sequenced fragment (5 exons), 7 known loci and one novel mutation named G4810372T (SNP8), were found in the fifth exon. In addition, we found 7 nonsynonymous substitutions. The allele frequency of only one SNP, g.4810191C > T (SNP1), was significantly different (*P* < 0.05) between case and control groups. The genotype frequencies of SNP1 (g.4810191C > T) and SNP3 (g.4810257G > T) were of significant difference between the case and the control groups (*P* < 0.05). Collectively, SNPs of the* MyD88* gene were significantly associated with susceptibility to Salmonella Pullorum infection, which can be used as a disease-resistant marker in chicken. These results provided a theoretical basis for future research on chicken breeding by marker-assisted selection.

## 1. Introduction

Salmonella Pullorum is a typical bacterial disease that has threated the modern poultry industry over the past years. Chicken becomes the carrier in the spread of Salmonella Pullorum and may cause economic losses worldwide through mortality, morbidity, and reductions in egg production [1]. Vaccination, antibiotics, and other drugs are the major preventive measure, but antibiotics will cause resistance to pathogens and antibiotic residues in poultry products and other issues. Generations of purifying elimination has been applied in domestic chicken populations, which would largely decrease genetic resistance of Salmonella Pullorum. In vertebrates, two types of immunity are developed to protect the host from infections: innate and adaptive [2]. The innate immune system is the first line of defense against microbial pathogens and of key importance early in bacterial infections. During infection, host inflammatory reaction is mediated by the recognition of pattern-recognition receptors (PRRs), which was activated by pathogen-associated molecular patterns (PAMPs) [3]. The TLR family is a major class of PRRs, which is critical for induction of the immune response to the given microbial challenge [4]. After recognized PAMPs, the TLR signaling pathway can be segregated into two specific ways: the MyD88-dependent pathway and the MyD88 independent pathway [5, 6]. The first pathway results in the activation of the nuclear factor-*κ*B (NF-*κ*B), and the expression of proinflammatory genes like tumor necrosis factor (TNF) and interleukin-1 (IL-1) [7, 8]. The second one upregulates interferon 3 (IRF3-) mediated expression of type I interferons (IFN) and IFN-inducible genes. The MyD88-dependent pathway is prominent for all TLRs except TLR3 [9]. As a universal adaptor protein, MyD88 plays an important role in activating the innate immune system [10]. The typical structure composed of amino terminal death domain of MyD88 recruits the downstream immune molecules, binding interactions with Interleukin-1 receptor-associated kinases (IRAKs) [11]. Some study on the relationship between TLR4 signaling pathway including related molecule and the susceptibility to disease showed that the signal pathways have played a major role in the infection [12, 13]. The expression of* TLR4* and immune related genes, such as* Gal 1*,* Gal 2*,* IL-8, IL-18,* and* IFN-γ*, established different degree of correlation against salmonella in hens [14].

In large yellow croaker,* MyD88* was reported to play a crucial role in defensing against pathogenic infection for its extremely differential expression between spleen and muscle [15, 16]. The lack of MyD88 protein in mice may result in susceptibility to* leishmania major* infection [17]. Though some studies broadly reveal the function of* MyD88* in TLR signaling pathways [18], the relationship between polymorphisms of the* MyD88* gene and Pullorum infection in chicken has not been reported. In view of the importance of the* MyD88* gene, it is necessary to analyze its sequence variations and to study whether its polymorphisms are associated with intersubject differences against salmonella. In this study, we selected chickens infected and uninfected with Salmonella Pullorum to conduct an association analysis with* MyD88*, aiming to provide a theoretical reference for poultry marker-assisted selection.

## 2. Materials and Methods

### 2.1. Pullorum Detection and Sample Collection

Based on case-control design, the method of whole blood glass plate agglutination (SN/T 1222-2003, AQSIQ) was used to test infection with Salmonella Pullorum. Whole blood sample (20 *μ*L) was mixed with Pullorum reagent (20 *μ*L) on a glass slide that was kept undisturbed for 3 min at room temperature (25 to 30°C). With the naked eye, agglutinations (clumping of RBCs) were read in infected samples. The level of infection have divided into “+++,” “++,” and “+” for 100%, 75%, and 50% aggregation, while no agglutination was read in uninfected ones (“−”). Grouped chickens were marked with “+++,” “++,” and “+” into the case.

Following common laying hen immunization program, chicken were vaccinated timely of immunization. 4,334 Er-lang mountainous hens have been tested at the time of 300 days of age, among which 128 infected subjects were collected as the cases and 163 uninfected subjects were collected as controls. The protocol was approved by the Committee on the Care and Use of Laboratory Animals of the State-Level Animal Experimental Teaching Demonstration Center of Sichuan Agricultural University. Blood samples were stored at −20°C.

### 2.2. DNA Extraction and DNA Pool Construction

The genomic DNA of all the samples was extracted by using the standard phenol/chloroform method. DNA samples were diluted or enriched at the level of (100 ± 3) ng/*μ*L with Nano Drop (ND-2000, Thermo Scientific) for homogeneity of DNA pool. A case DNA pool and a control one were composed of 30 samples that each contains 2 *μ*L DNA selected at random, respectively.

### 2.3. Primer Design

Using the Primer Premier 5 software (Premier BioSoft, Palo Alto, CA, USA), five pairs of primers were designed to cover the entire coding region (CDS) of the* MyD88* gene (Table 1) from* Gallus gallus* (GenBank accession number NM_001030962).

### 2.4. PCR Amplification and Sequencing

Polymerase chain reaction (PCR) amplification was performed in a volume of 25 *μ*L reaction mixture containing 50–100 ng of DNA, 0.3 *μ*M of each forward and reverse primer, and 15 *μ*L of 2x Taq PCR MasterMix (Tiangen Biotech Co., China). The procedure was carried out with one cycle of denaturalization at 94°C for 5 min, 35 cycles of 94°C for 40 s, appropriate annealing temperature (Table 1) for 30 s, and 72°C for 40 s, followed by a final extension cycle at 72°C for 8 min. All PCR products of DNA pool and individual were directly sequenced by the Shenzhen BGI Biotechnology Company (Beijing, China).

### 2.5. Statistical Analysis

The sequencing electrophoretogram was read by Chromas software. Sequence variations, composition, and variable sites were identified using DNAstar software (DNAstar Inc., Madison, WI, USA). Hardy-Weinberg equilibrium, pairwise linkage disequilibrium (*D*′), and association analysis were conducted by Haploview software (version 3.32, http://www.broad.mit.edu/mpg/haploview/). Complementary analysis, such as the standard Chi-squared test of genotype frequency, was performed by use of R software (version 3.0.2, The R Foundation for Statistical Computing).

## 3. Result

### 3.1. Sequence Variations in CDS Region of the* MyD88* Gene

No mutation was observed in* MYD88* CDS region except for the exon5. A total of 8 SNPs were detected in exon5, including 7 known SNPs (http://www.ncbi.nlm.nih.gov/projects/SNP/) and a novel mutation locating at 4810372 bp of the chicken genome named G4810372T (SNP 8). 7 SNPs (SNP1, SNP2, SNP3, SNP4, SNP5, SNP6, and SNP8) were nonsynonymous variants leading to amino acids changes, while the allele change of SNP7 leads to a synonymous mutation (Table 2).

### 3.2. The Hardy-Weinberg Equilibrium

The Hardy-Weinberg equilibrium tests of the 8 SNPs in the case and control group were shown in Tables 2 and 3. The observed heterozygosity of all SNPs was at a general level as expected. Most of SNPs fit the assumption of the Hardy-Weinberg equilibrium except SNP2 (*P* < 0.05) that was removed from the analysis. The minor allele frequencies (MAF) of all the mutations were more than 0.01.

### 3.3. Allele and Genotype Frequency of the Mutated Loci

The results of the allele and genotype frequency of the 8 SNPs in the case and control groups were shown in Tables 3 and 4. The Chi-squared test was used to compare the allele frequencies in the* MyD88* gene between the case and control groups. The data showed that only SNP1 (*χ* ^2^ = 4.604, *P* = 0.0319) was significantly correlated with Salmonella Pullorum at the allelic level. At the genotype level, two SNPs, SNP1 (*χ* ^2^ = 7.924, *P* = 0.019, OR = 0.6752, and 95% CI = 0.4712–0.9674) and SNP3 (*χ* ^2^ = 8.353, *P* = 0.015, OR = 0.7555, and 95% CI = 0.5290–1.079), showed significant correlations with Salmonella Pullorum in the chicken population.

### 3.4. Association between Haplotypes and Susceptibility to Pullorum

**Figure 1:**
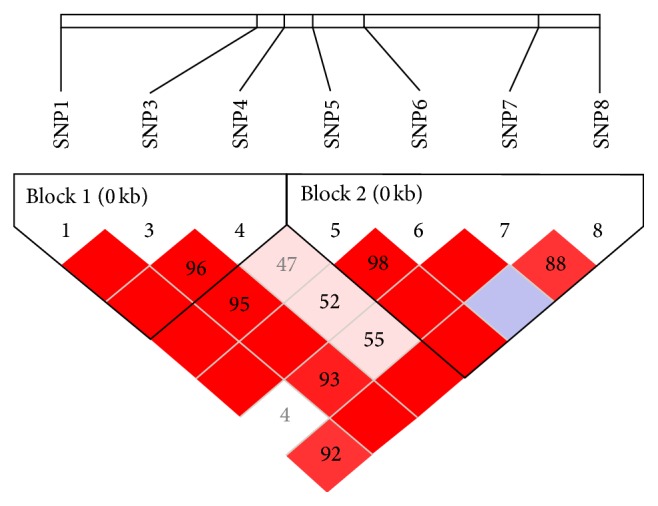
LD value within each diamond represents the correlation between pairs of SNPs (measured as *D*′) in the CDS of the* MyD88* gene. The diamond without a number means complete LD (*D*′ = 1). Darker red of the diamonds indicates higher *D*′, while white indicates lower *D*′.

Figure 1 revealed that the degree of the linkage disequilibrium (LD) indicates the correlation between polymorphic variants at different positions in the* MyD88* gene. With 2 blocks in dark red in the *D*′ plot, it was clear that the SNPs of block 1 (SNP1, SNP3, and SNP4) and block 2 (SNP5, SNP6, SNP7, and SNP8) are of high *D*′, respectively. Nevertheless, SNP6 and SNP8 were in equilibrium and independent of one another in block 2. Haplotype analysis showed that the haplotype groups CTT (*χ* ^2^ = 4.604, *P* = 0.0319) and TTC (*χ* ^2^ = 11.643, *P* = 6.0*E* − 4) in block 1 of the* MyD88* gene correlated significantly with resistance to Salmonella Pullorum infection (Figure 1, Table 5). Haploid type distribution of block 2, composed by SNP5, SNP6, SNP7, and SNP8, was not significantly different (*P* > 0.05) in the case and control groups. Haploid types in block 2 had no relationship with disease resistance.

## 4. Discussion

Infectious disease has an effect on the food safety and spreads broadly in chicken, especially in commercial lines. In the past years, the contradiction between antibiotics abuse and food safety has increasingly grown [19, 20]. In addition, selection based heavily on production performance could disadvantageously affect individual immunity leaving chickens less resistive to pathogenic bacteria [21]. Recently, the intersubject differences in immunity that associated with missense mutations in innate immune genes, such as major histocompatibility complex (*MHC*) [22, 23] and myxovirus resistance gene (*Mx*) [24], have significantly increased in chicken. Consequently, studying the basic knowledge of mutations in innate immune genes has an important significance for chicken disease-resistant breeding.

In previous study, the use of SNP of innate immune genes, such as natural resistance associated macrophage protein 1 (*Nramp1*) [25],* TLR4* [26],* CD28,* and* MD-2 *[27], leads to enhancement of Salmonella Pullorum resistance in chicken. Yet, in another study, SNP797T/C genotype in the first intron of the* MyD88* gene in swine was of no significant correlation with resistance to Salmonella Pullorum infection [28]. Susceptibility to Salmonella Pullorum infection showed the differences across species [29]. Through the analysis, the polymorphisms of the CDS area in* MyD88* gene and the correlation with resistance to salmonella suggested that* MyD88* gene may be one of the major Salmonella Pullorum resistant genes in innate immune system.

The Hardy-Weinberg equilibrium is influenced by many factors, including selection, the rate of recombination, the rate of mutation, genetic drift, the system of mating, population structure, and genetic linkage. Due to the selection and foreign blood imported in the chicken population, the alleles and genotypes of SNP2 loci were unstable and SNP2 was removed from statistic analysis. We screened out a newly identified SNP site named G4810372T (SNP 8), and the follow-up studies may be needed to test the functional significance for the newly identified SNP site [30].

In genetics, a missense mutation, a type of nonsynonymous substitution, results in truncation of the resulting protein and protein nonfunctional. According to the Human Gene Mutation Database (HGMD), missense mutation of* CRYBB2* leads to congenital cataract in a family of Croatian origin [31]. In the CDS of* MyD88*, there are 376 amino acid residues [32], 7 of which have changed for they have underwent nonsynonymous substitutions. The homology of DNA sequence (1122/1130) was presented higher than the amino acid sequence homology (369/376). In other words, the* MyD88* gene polymorphism was of higher performance on the amino acid levels. All the mutations locating only in extron 5 declared that* MyD88* gene was highly conserved. In analysis by the SMART software (http://smart.embl-heidelberg.de/), the 7 missense mutations locate out of the typical structure of* MyD88*, the N-terminal death domain, C-terminal toll-interleukin-1 receptor (TIR) domain, and intermediate domain. Therefore, the 7 substitutions of amino acid caused by polymorphisms of the* MyD88* gene may have the potential of resistance to bacteria and pathogens.

To reveal the relationship between polymorphisms of the* MyD88* gene and disease resistance, we conducted a Mendelian population-based case-control study in chicken [33]. The OR and 95% confidence interval are used to determine the resistance or susceptibility to Pullorum infection. The OR value less than 1 means resistance effect, while value greater than 1 means susceptibility effect [34]. At the same time, the *P* values determine the significance of its association. SNP1 alleles distribution, of the lowest OR value (OR = 0.6752), made significant differences in case and control samples (*P* < 0.05). The genotypes distribution of SNP3 and SNP1 revealed significant differences in case and control samples (*P* < 0.05). Results indicated that polymorphisms of the* MyD88* gene and susceptibility to Pullorum infection have significant correlation.

In the study of the multiple loci in linkage disequilibrium (LD) and the correlation of disease, the correlation of haploid types is more effective than a single locus analysis [35]. Many phenotypic traits are often the result of the interaction between multiple loci, especially in a haploid type block, and caused by interaction among a set of mutations in a certain area of the chromosome [36, 37]. In the correlation analysis of haploid type, the first thing is to determine the strength of LD (*D*′) and type in the group. LD exists widely in the family of chicken population in this study. The results showed that the SNPs among block 1 and block 2 groups were in strong linkage disequilibrium state (*r* ^2^ > 0.33, |*D*′ | > 0.8). Containing the analysis results of SNP1 loci, difference between haploid type composed by SNP1, SNP3, and SNP4 loci and resistance to disease was very significant. The result showed that the analysis of haploid types had better statistical effect (*P* < 0.01). Effects between SNPs loci in haploid type set canceled each other out on account of interactive effects of genes, leading to no significant difference between SNP5, SNP6, SNP7, and SNP8 loci haploid type and disease resistance.

In conclusion, after comparison and analysis of the genetic variation of* MyD88* gene, we found that the novel mutation G4810372T may have an effect on the individual immune. But further functional studies are necessary to evaluate the molecular mechanism caused by polymorphisms of the* MyD88* gene. The correlation analysis of polymorphisms of the* MyD88* gene and susceptibility of Salmonella Pullorum in chicken showed that, in each mutation, alleles in SNP1 locus and genotypes of SNP1 and SNP3 had a significant effect against salmonella. What is more, the advantaged haploid type (TTC) combined by SNP1, SNP3, and SNP4 loci played a very significant role in genetic resistance to Salmonella Pullorum infection. Polymorphisms of the* MyD88* gene or advantaged haploid type in a particular area had a certain positive effect against susceptibility to Pullorum infection. From the above, the* MyD88* gene can be used as a candidate gene for follow-up study, which could provide a theoretical reference for poultry marker-assisted selection.

## Figures and Tables

**Table 1:** The primer sequences to amplify the CDS of *MyD88* gene in chicken.

Name	Amplicon size (bp)	Sequence (5′-3′)	Production (bp)	Anneal temperature (°C)	Region
P1	19	F: GGCTCCTTCCAACCCAAAC	793	59.0	exon1
	21	R: AGACCGATCTCACCTCACCAC			

P2	22	F: CAAGAAGGCACTGGGTAAACTC	483	54.6	exon2
	21	R: GAATAGGCAACGGGAAGAATG			

P3	20	F: TCTGTCAAAGGCTGGGGAAG	297	54.0	exon3
	19	R: ACACTGAGCTGCCCCAAGC			

P4	24	F: GTTCCTGCTCAACCACAACTAAAG	443	52.5	exon4
	22	R: GGGTTCTGGTTCAGTAGGCATC			

P5	23	F: TAGAAGCAGGATGTGAGTGTGGC	501	59.6	exon5
	25	R: GCAGTGACTCAGTCTTTAAGCGAAT			

**Table 2:** Change of alleles and amino acids in the CDS region of *MyD88* gene.

Markers	ID	Position	Obs HET	Expt HET	Allele change	Amino acids change	Mutation
SNP1	rs317890917	4810191	0.457	0.412	C>T	307 Leucine > phenylalanine	Nonsynonymous
SNP2	rs14131328	4810253	0.258	0.349	G>C	327 Glutamine > Histidine	Nonsynonymous
SNP3	rs14131329	4810257	0.405	0.419	G>T	329 Valine > Leucine	Nonsynonymous
SNP4	rs14131330	4810266	0.481	0.481	T>C	332 Cysteine > Arginine	Nonsynonymous
SNP5	rs14131331	4810276	0.436	0.483	G>A	335 Arginine > Histidine	Nonsynonymous
SNP6	rs14131332	4810293	0.471	0.477	G>C	341 Glycine > Arginine	Nonsynonymous
SNP7	rs14131333	4810352	0.474	0.483	A>G	360 Leucine > Leucine	Synonymous
SNP8	G4810372T	4810372	0.072	0.082	G>T	367 Serine > Isoleucine	Nonsynonymous

Obs HET is observed heterozygosity; Expt HET is expected heterozygosity.

**Table 3:** Allele frequency of mutation loci in the *MyD88* gene.

Markers	Alleles		*χ* ^2^, *P* value	OR	95% CI	HWE (*P*)	MAF
SNP1 (rs317890917)	T	C	*χ* ^2^ = 4.604 *P* = 0.0319	0.6752	0.4712–0.9674	0.0873	0.29
Cases	170 (0.664)	86 (0.336)					
Controls	243 (0.745)	83 (0.255)					

SNP3 (rs14131329)	T	G	*χ* ^2^ = 2.384 *P* = 0.1226	0.7555	0.5290–1.079	0.6509	0.299
Cases	171 (0.668)	85 (0.332)					
Controls	237 (0.727)	89 (0.273)					

SNP4 (rs14131330)	T	C	*χ* ^2^ = 0.2487 *P* = 0.6180	1.0888	0.7794–1.521	1.0	0.402
Cases	156 (0.609)	100 (0.391)					
Controls	192 (0.589)	134 (0.411)					

SNP5 (rs14131331)	G	A	*χ* ^2^ = 0.3047 *P* = 0.5810	1.098	0.7869–1.534	0.122	0.407
Cases	155 (0.605)	101 (0.395)					
Controls	190 (0.583)	136 (0.417)					

SNP6 (rs14131332)	G	C	*χ* ^2^ = 0.0155 *P* = 0.9009	1.022	0.7306–1.4282	0.8877	0.393
Cases	156 (0.609)	100 (0.391)					
Controls	197 (0.604)	129 (0.396)					

SNP7 (rs14131333)	G	A	*χ* ^2^ = 0.3166 *P* = 0.5736	0.9089	0.6517–1.2677	0.8152	0.409
Cases	148 (0.578)	108 (0.422)					
Controls	196 (0.601)	130 (0.399)					

SNP8 (G4810372T)	T	G	*χ* ^2^ = 0.1708 *P* = 0.6794	0.8445	0.3786–1.884	0.1766	0.043
Cases	244 (0.953)	12 (0.047)					
Controls	313 (0.960)	13 (0.040)					

CI, confidence interval; OR, odds ratio; HWE (*P*), *P* value of the Hardy-Weinberg equilibrium test; and MAF, minimum allele frequency.

**Table 4:** Genotype frequency of mutation loci in the *MyD88* gene.

Markers	Genotypes			*χ* ^2^, *P* value
SNP1 (rs317890917)	TT	TC	CC	*χ* ^2^ = 7.924 *P* = 0.019
Cases	50 (0.391)	70 (0.547)	8 (0.072)	
Controls	90 (0.552)	63 (0.387)	10 (0.061)	

SNP3 (rs14131329)	TT	TG	GG	*χ* ^2^ = 8.353 *P* = 0.015
Cases	54 (0.422)	63 (0.492)	11 (0.086)	
Controls	92 (0.564)	53 (0.325)	18 (0.110)	

SNP4 (rs14131330)	TT	TC	CC	*χ* ^2^ = 3.2 *P* = 0.202
Cases	44 (0.344)	68 (0.531)	16 (0.125)	
Controls	60 (0.368)	72 (0.442)	31 (0.190)	

SNP5 (rs14131331)	GG	GA	AA	*χ* ^2^ = 2.174 *P* = 0.337
Cases	47 (0.367)	61 (0.477)	20 (0.156)	
Controls	62 (0.380)	66 (0.405)	35 (0.215)	

SNP6 (rs14131332)	GG	GC	CC	*χ* ^2^ = 2.805 *P* = 0.246
Cases	45 (0.352)	66 (0.516)	17 (0.133)	
Controls	64 (0.393)	69 (0.423)	30 (0.184)	

SNP7 (rs14131333)	GG	GA	AA	*χ* ^2^ = 2.999 *P* = 0.223
Cases	40 (0.313)	68 (0.531)	20 (0.156)	
Controls	63 (0.387)	70 (0.429)	30 (0.184)	

SNP8 (G4810372T)	TT	TG	GG	*χ* ^2^ = 2.843 *P* = 0.241
Cases	118 (0.922)	8 (0.063)	2 (0.016)	
Controls	150 (0.920)	13 (0.080)	0 (0)	

**Table 5:** The haplotype analysis of 8 *MyD88* SNPs.

	Haplotype groups	Frequency (cases)	Frequency (control)	*χ* ^2^	*P* value
Block 1	TTT	0.273	0.325	1.818	0.1776
	TGC	0.332	0.263	3.222	0.0727
	CTT	0.336	0.255	4.604	0.0319
	TTC	0.059	0.148	11.643	6.0*E* − 4

Block 2	ACGT	0.383	0.396	0.1	0.7515
	GGAT	0.380	0.359	0.272	0.6023
	GGGT	0.171	0.184	0.17	0.6803
	GGAG	0.042	0.040	0.016	0.8979
	AGGT	0.012	0.022	0.805	0.3695
